# Demulsification of asphaltene stabilized crude oil emulsions by biodegradable ethylcellulose polymers with varying viscosities

**DOI:** 10.1038/s41598-023-27973-x

**Published:** 2023-01-19

**Authors:** Arafat Husain, Ahmad A. Adewunmi, Afeez Gbadamosi, Mamdouh A. Al-Harthi, Shirish Patil, Muhammad Shahzad Kamal

**Affiliations:** 1 Department of Chemical Engineering, King Fahd University of Petroleum and Minerals, Dhahran, 31261 Saudi Arabia; 2 Center for Integrative Petroleum Research, King Fahd University of Petroleum and Minerals, Dhahran, 31261 Saudi Arabia; 3 Department of Petroleum Engineering, College of Petroleum and Geosciences, King Fahd University of Petroleum and Minerals, Dhahran, 31261 Saudi Arabia

**Keywords:** Fossil fuels, Crude oil

## Abstract

Efficient demulsifiers for fast demulsification of asphaltene stabilized crude oil emulsions are currently in high demand. In this work, we evaluated the demulsification potential of ethyl cellulose (EC) demulsifiers with varying viscosities—4 cp, 22 cp, and 100 cp, designated as EC-4, EC-22, and EC-100. Demulsifcation efficiency (DE) of these demulsifiers to remove water from emulsions produced from distilled water, seawater, and different salts (NaCl, MgCl_2,_ and CaCl_2_) solution were assessed using the bottle test technique at ambient and elevated temperatures (25 °C and 90 °C). The bottle test outcomes showed that EC-4 and EC-22 had better performance at the ambient conditions to demulsify the emulsions formed from distilled water with %DE of 85.71% and 28.57%, respectively, while EC-100 achieved 3.9% water removal owing to its high viscosity which inhibited its adsorption at the oil–water interface. At demulsification temperature (90 °C) under the emulsions from distilled water, the %DE of EC-4, EC-22, and EC-100 was 99.23%, 58.57%, and 42.85%, respectively. Seawater hastened the demulsification activities of these demulsifiers. Also, these demulsifiers demonstrated excellent demulsification in emulsions from various salts. The demulsification performance of the EC-4 demulsifier in the presence of any of these salts was approximately 98% while MgCl_2_ and CaCl_2_ accelerated the water/oil separation performance of EC-22 and EC-100 by promoting their diffusion and adsorption at the interface. Viscosity and shear stress measurements corroborated the results obtained from the bottle tests. Injection of EC demulsifiers led to a reduction in the viscosity and shear stress of the formed emulsion. Reduction in the shear stress and viscosity were highest in EC-4 and lowest in EC-100. Optical microscopic images of emulsion injected with EC-4 demulsifier were analyzed at various periods during viscosity measurements. Based on the optical images obtained at different durations, a demulsification mechanism describing the activity of the EC demulsifier was proposed.

## Introduction

One of the major setbacks faced by the petroleum industry during the production and transportation of crude oil is the formation of highly stable and complex emulsions in association with saline water^1^. During the production of crude oil, the emulsion formed can be quite complex and can be categorized as two or three-phase emulsions. The most commonly encountered among these emulsions is the water-in-oil (W/O) emulsion, whereas, the others are encountered rarely^2^. Generally, crude oil produced from reservoirs contains a small fraction of impurities such as water, salts, and sediments. The formation of the homogenous emulsion from two immiscible liquids is attributed to the applied shear force and pressure on the mixture during the initial stages of production and essentially the presence of natural emulsifiers in crude oil helps in stabilizing the emulsion^3^.

The molecules of the natural emulsifying agent act by migrating towards the oil and water interface where they form an interfacial resistance surrounding the water droplet thus inhibiting the coalescence of the water droplets. These naturally existing emulsifying agents in crude oil are mainly asphaltenes, resins, wax, and solids^4^. Saline water will cause serious problems, such as catalyst poisoning in downstream refinery units, corrosion, and fouling in pipelines and equipment^5^. Pipelines that suffer from fouling undergo a reduction in active diameter that results in elevated pressure drop and handling cost. In addition to all these, the accumulation of water causes a reduction in the quality of crude oil thus affecting its physical and thermal properties. Thus, emulsion breaking and water separation from crude oil are necessary. Therefore, water separation from crude oil or demulsification of the emulsion becomes a necessity. Methods to demulsify emulsions are broadly classified into four categories namely chemical, mechanical, thermal, and electrical processes. According to the current trends, chemical demulsification is one of the most effective and economical methods to disintegrate crude oil emulsion^6^.

The selection of an effective emulsion-breaking agent with respect to the chemical configuration of the crude oil is one of the main hurdles during the crude oil desalting processes^7^. Salt precipitation^8^ and corrosion control^9^ are very crucial in the selection of appropriate chemical demulsifiers. A lot of research work focusing on the chemical demulsification of crude oil has been carried out and is still in progress to develop efficient and environmentally friendly chemical demulsifiers^10–12^. Many studies related to ethylene oxide-propylene oxide (EO-PO) block copolymers have been conducted for efficient demulsification. One such study was conducted by Al-Sabagh^13^ with five polymeric surfactants involving different formulations of EO-PO ratios and their effect on demulsification efficiency (DE) of W/O crude oil emulsion was investigated. The results of their investigation suggested that the increased DE was a result of the increased molecular weight of the demulsifier. Zaki et al.^14^ studied the effect of hydrophobic lipophilic balance (HLB) on the demulsification efficiency of W/O emulsion for polypropylene oxide (PPO)-polyethylene oxide (PEO) copolymers. The results attained indicated an increase in HLB values for efficient demulsification as a result of an increase in the amount of hydrophilic ethylene oxide. A study by Follotec et al.^15^ focused on linking DE of triblock copolymer demulsifier consisting of equally distributed hydrophilic tails polyethylene oxide (PEO) and central hydrophobic part of polydimethyl siloxane (PDMS) to the PEO to PDMS ratios. The results pointed out that most of the hydrophobic agents were marked with undesirable demulsification performance, while higher demulsification tendencies were observed for hydrophilic copolymer agents in parallel with changing PEO/PDMS ratio. Additionally, a mechanism was suggested to forecast the coalescence and flocculation of water droplets in W/O emulsion. Similarly, the modeling approach has been used to evaluate the simultaneous effects of key parameters affecting the demulsification performance of chemical demulsifiers^16,17^.

Ethylcellulose (EC) has been suggested as a potential alternative biodegradable and non-toxic chemical demulsifier. Ethylcellulose (EC) was tested by Feng et al.^18,19^ to break naphtha-diluted bitumen emulsion. These authors proposed a mechanism for emulsion breaking. With 90% removal of water from the bitumen-diluted emulsion, EC served as an effective demulsifier. The mechanism described was a combination of coalescence and flocculation of water droplets through competitive adsorption of EC at the oil–water interface by disturbing the protective film layer of the water-bitumen interface. In a study conducted by He et al.^20^, EC and EO/PO copolymers were applied for the demulsification of water-bitumen emulsion based on a dynamic liberation test and contact angle. The results indicated that both polymeric demulsifiers showed good potential as demulsifying chemicals for breaking stable emulsions. To further explore the activity of EC as a demulsifier, the current study aimed to investigate how some EC demulsifiers with varying viscosities impact the demulsification of heavy crude oil emulsions stabilized by asphaltenes. To the best of our knowledge, no study has been conducted on the assessment of how the viscosity of EC can affect the demulsification activity. Herein, the demulsification efficiencies of EC demulsifiers having unique viscosities were examined by producing emulsions from crude oil and using either distilled water, seawater, or different salt solutions. Preliminary demulsification investigations at ambient and elevated temperatures revealed that differing viscosity of the examined EC demulsifiers had a huge impact on their demulsification performance and also affect their migration and adsorption at the oil–water interface. Following this, the ability of the investigated EC demulsifiers to diffuse and adsorb at the oil–water interface and rupture the asphaltene molecules was further assessed by measuring the shear stress and viscosities of the formulated emulsions before and after the injection of each demulsifier. Optical microscopic analysis was conducted during viscosity measurement to examine the transition of emulsion breaking sequence in the presence of the EC demulsifier. Certainly, an active demulsifier should be a type that would disrupt the emulsifying substances at the oil–water interface. The EC demulsifiers employed herein are biodegradable, cost-effective, and promising chemicals for smart oil–water separation in the petroleum industry.

## Materials and methods

### Materials

The crude oil used is obtained from a local oilfield. The SARA and physical parameters of this crude oil are tabulated in Table 1, and a detailed procedure describing its characterizations has been reported elsewhere^21^. The W/O emulsion was formed by using either distilled water, different salts (MgCl_2_, NaCl, and CaCl_2_) solutions, or seawater. The ions found in seawater is provided in Table 2. Subsequently, the W/O emulsion will be expressed as crude oil emulsion or emulsion throughout this current report. Three EC chemical demulsifiers designated as EC-4, EC-22, and EC-100, having viscosities of 4cP, 22cP, and 100cP, respectively, were employed for demulsification activities. These demulsifiers are identical in terms of structure and density but possessed different viscosities as reflected in Table 3.

**Table 1:** Physical characteristics of the used crude oil^22^.

Physical characterization/Mass percentage	Value
API specific gravity @ 15 °C	0.86
API gravity @ 15 °C	32.49
Density (g/cm^3^) @ 15 °C	0.87
Viscosity (mPa s) @ 15 °C	10.9
**SARA fractions**	
Saturates (wt%)	36.2
Asphaltenes (wt%)	2.8
Resins (wt%)	11
Aromatics (wt%)	50

**Table 2:** The composition of the prepared seawater^22^.

Ions	Seawater conc (mg/l)
Na^+^	18,300
Ca^2+^	700
Mg^2+^	2100
${\text{SO}}_{4}^{2-}$	4300
Cl^-^	32,200
${\text{HCO}}_{3}^{-}$	100
Total	57,700

**Table 3:** Viscosity and structure of ethyl cellulose (EC) demulsifiers used.

Demulsifier	Viscosity (cp)	Density (g/cm^3^)	Molecular structure
EC-4	4	1.14	
EC-22	22	1.14	
EC-100	100	1.14	

### Preparation of emulsion

Crude oil emulsions were prepared at ambient conditions. The preparation method involved mixing crude oil and water (either distilled water, different salt solutions, or seawater) in a beaker with water to oil ratio of 7:3 and mixing them with a stirrer at a constant speed of 1500 rpm. Water was added gradually into the crude oil and agitated for 40 min until the homogenous phase was achieved. Each EC demulsifier was prepared by mixing in methanol solvent to enhance diffusion when injected into the emulsion. Demulsifier and methanol mixture was formed, in which the quantity of each demulsifier was maintained at 10 wt%. The emulsion preparation procedure followed in this study has been reported elsewhere^23,24^.

### Demulsification test

Demulsification tests were carried out at ambient and elevated temperatures (25 °C or 90 °C) to evaluate the effect of every EC demulsifier to break the prepared emulsion and separate oil and water into distinct phases. The required concentration of demulsifier was added into the emulsion in the test tube and hand shaken for 20 s to get proper mixing of the added demulsifier. The demulsification efficiency (DE) was estimated via the percentage of water removal from the crude oil emulsion using formula^25^.

1 $$\text{\% DE = }\frac{\text{Separated\,\, water\,\, (ml)\,\, with\,\, respect \,\,to \,\,time}}{\text{Initial \,\,total \,\,water\,\, (ml)\,\, in\,\, emulsion }}{\times 100}$$

### Characterizations

The emulsion droplet sizes were analyzed using the Leica DM2000 backed by a highly efficient light-emitting diode (LED). Shear stress and viscosity measurements were determined using the discovery hybrid rheometer manufactured by TA Instruments. For every experimental run, a minimum of 23 ml emulsion was injected into a concentric cylinder geometry. The measurements were conducted at 25 °C to eliminate the negative effect of evaporation which may cause data inconsistencies. The parameters under the observations were the viscosity and shear stress of the formulated emulsion before and after the addition of the EC demulsifiers.

## Results and discussion

### Performance of EC demulsifier

**Figure 1:**
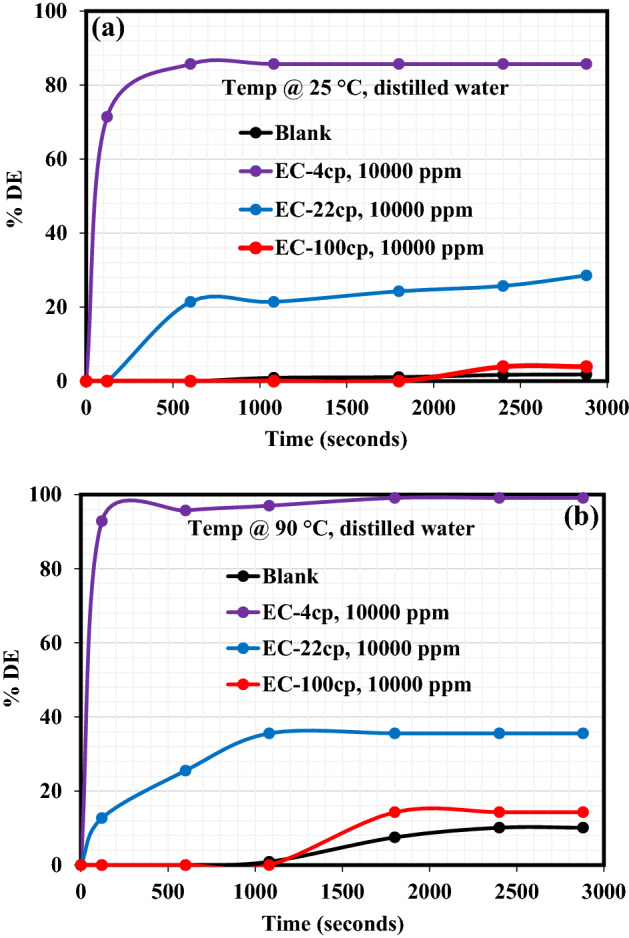
Demulsification performance of EC demulsifiers resulting from crude oil emulsion formed with distilled water at: (**a**) 25 °C and (**b**) 90 °C.

**Figure 2:**
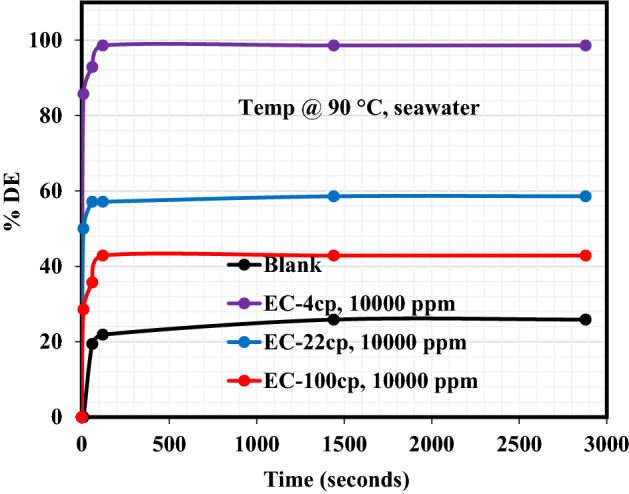
Demulsification performance of EC demulsifiers resulting from emulsion formed with seawater at 90 °C.

The Demulsification performance of EC-4, EC-22, and EC-100 to break the crude emulsions formulated from distilled water and seawater is reported in this section. The demulsification activity was carried out inside the oven at ambient and elevated temperatures (25 °C and 90 °C). Figure 1a exhibits the demulsification efficiency (DE) plot at 25 °C of each EC to demulsify the crude oil emulsion formulated from the distilled water. As can be seen from these plots, the %DE of EC-4 and EC-22 to break the emulsion and cause water removal was very rapid for about 600 s and both demulsifiers’ demulsification activity remained almost constant from the remaining demulsification periods. EC-4 and EC-22 exhibited the DE of 85.71% and 28.57%, respectively, after 2880 s of the demulsification test. On the other hand, oil/water separation was sparingly encountered in the case of the blank sample and EC-100 demulsifier, until around 2000 s when the blank sample and EC-100 achieved a little oil/water separation. The %DE of the blank sample and EC-100 after 2880 s of demulsification activity was 1.8% and 3.9%, respectively. It is believed that the high viscosity of this demulsifier (EC-100) retarded its diffusion and adsorption at the oil–water interface, hence, being unable to break the asphaltene molecules. Also, since EC is hydrophobic^26^ in nature, the EC-100 demulsifier probably settled in the crude oil with little or no diffusion during demulsification activity at ambient conditions. Following the demulsification activity at 90 °C as shown in Fig. 1b, the demulsification performance of these EC demulsifiers changed. The %DE of blank sample and EC-4, EC-22, and EC-100 demulsifiers after 2880 s were 10.1%, 99.1%, 35.58%, and 14.29%, respectively, indicating the improved performance of these demulsifiers at elevated temperatures. Although, at the initial stage of the demulsification process, the EC-100 demulsifier failed to be active in separating water from the emulsion for a certain period. After 1080 s during the demulsification activity, the operating demulsification temperature (90 °C) is believed to have caused the EC-100 viscosity to be light enough to migrate, adsorb and break the asphaltenes at the oil–water interface. Figure 2 shows the plot elucidating the performance of EC-4, EC-22, and EC-100 demulsifiers to destabilize emulsion produced from seawater at 90 °C. The water/oil separation was very rapid at the initial demulsification period of 120 s and the separation remained constant afterward. Accordingly, the %DE of the reference sample (blank), EC-4, EC-22, and EC-100 after 2880 s demulsification period was 25.88%, 99.23%, 58.57%, and 42.85%, respectively. Undoubtedly, multiple salts found in seawater must have accelerated the activity of these demulsifiers and also prompted their diffusion, adsorption at the oil–water interface, and eventual destabilization of asphaltene molecules.

### Effect of salts on the performance of EC demulsifiers

**Figure 3:**
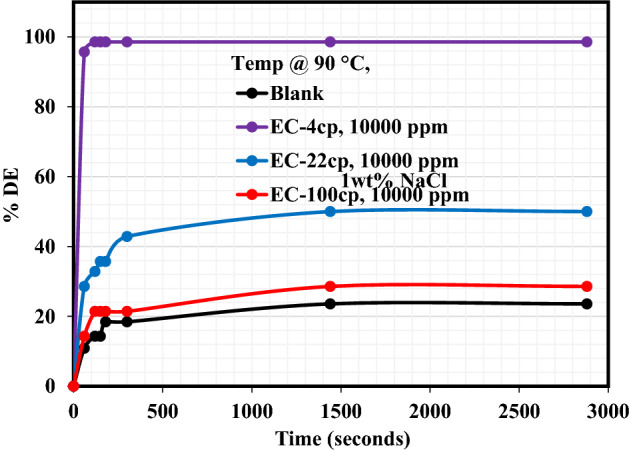
Effect of NaCl salt on the demulsification efficiency of EC demulsifiers.

**Figure 4:**
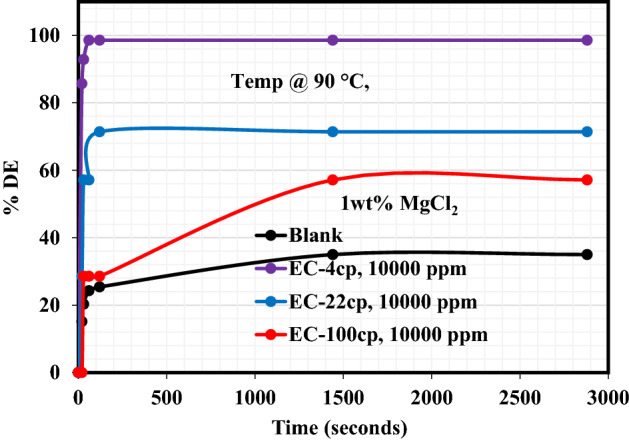
Effect of MgCl_2_ salt on the demulsification efficiency of EC demulsifiers.

**Figure 5:**
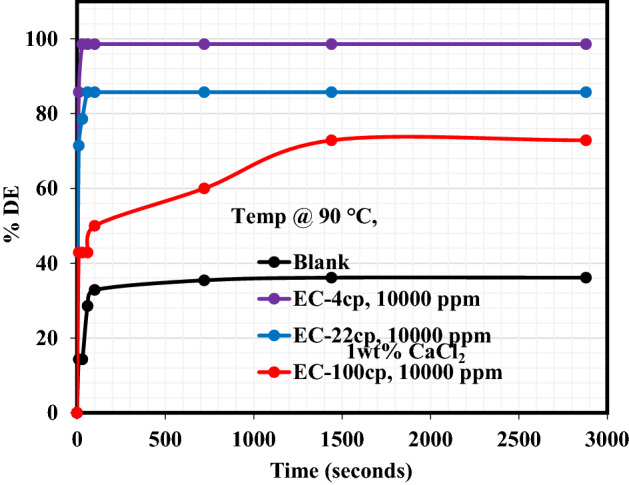
Effect of CaCl_2_ salt on the demulsification efficiency of EC demulsifiers.

Salt plays a decisive role in influencing the stability of emulsion as well as its interfacial properties. The addition of salt to the demulsifier alters the distribution of natural emulsifiers in the emulsion from the oil phase to the water phase^27^. The presence of a demulsifier in association with the inorganic salt performs two decisive roles. Firstly, reducing the repulsive forces between the ions by compression of the electric dual layer of the interface, therefore, enhancing the interaction and adsorption of surfactant at the interface. Secondly improving the hydrophilicity of the demulsifier thus making the micelle formation simpler and escape of the demulsifier from the bulk phase to the interface easy^28^. Figures 3, 4, 5 reveal the demulsification performance of these EC demulsifiers to destabilize the crude oil emulsions and bring about oil/water separation in the presence of 1 wt% of either NaCl, MgCl_2_ or CaCl_2_ salt. The impact of these salts on the demulsification performance of EC-4, EC-22, and EC-100 demulsifiers is better understood from the data tabulated in Table 4. As reflected in this table, the heating effect on the blank samples in the presence of NaCl, MgCl_2_ and CaCl_2_ salts led to substantial water removal, with highest water removal of 36.14% after 2880 s in the crude oil emulsion containing 1wt% CaCl_2_. The %DE of EC-4 demulsifier in the presence of NaCl, MgCl_2_ and CaCl_2_ salts was practically the same after 2880 s while the %DEs of EC-22 and EC-100 were distinct in the presence of these salts. It is very glaring that the presence of MgCl_2_ and CaCl_2_ salts had a pronounced effect on the demulsification activity of EC-22 and EC-100 demulsifiers as compared to the NaCl salt. The inference from these observations is that the divalent ions played a crucial role in the water/oil separation, by promoting the migration, adsorption, and interaction of EC-22 and EC-100 demulsifiers at the oil–water interface as compared to the monovalent ion in NaCl salt. Similar salt effects of inducing swift water–oil separation in the presence of demulsifiers have been reported in previous studies^23,24,29^^.^

**Table 4:** Effect of salts on the demulsification performance of EC demulsifiers at 90 °C.

Salts	%DE of EC Demulsifiers @ 90 °C			
	Blank @ 2880 s	EC-4 @ 2880 s	EC-22 @ 2880 s	EC-100 @ 2880 s
1wt% NaCl	23.57	98.57	50	28.57
1wt% MgCl_2_	35.01	98.77	71.42	57.14
1wt% CaCl_2_	36.14	98.59	85.71	72.86

### Emulsion viscosity with and without EC demulsifier

**Figure 6:**
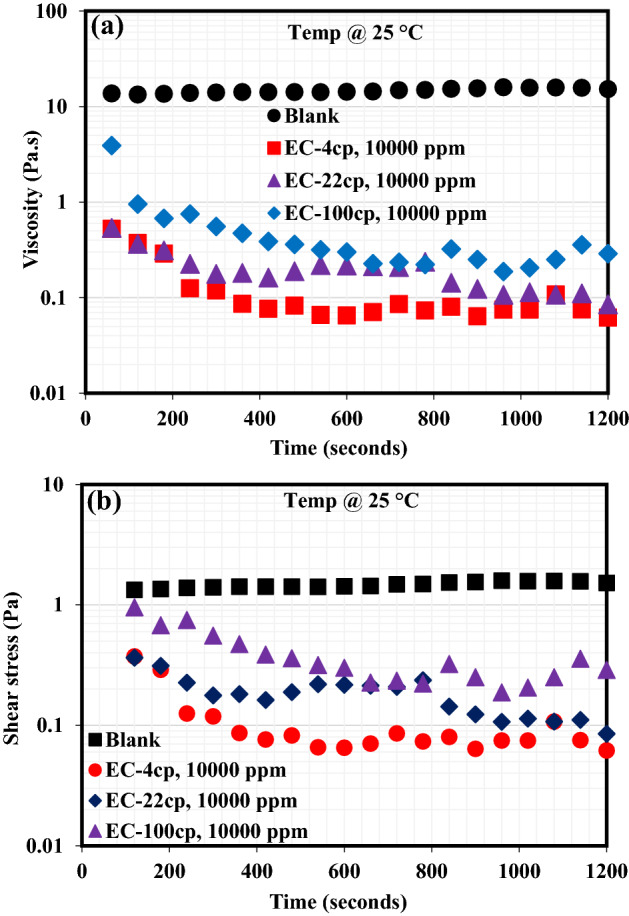
(**a**) Viscosity behavior and (**b**) Shear stress profile of emulsions with and without EC-4, EC-22 or EC-100 demulsifiers.

The significance of the demulsifying additive is to rupture the crude oil emulsion stabilizing substance, specifically, asphaltenes and resin molecules. During this process, the inevitable thinning of the interfacial film at the oil–water interface occurs and eventually leading to its collapse, causing water droplets to coalesce and viscosity reduction. Also, the shear stress of a stable emulsion is expected to drop following the addition of an effective demulsifier. This pattern can be monitored by evaluating the viscosity and shear stress of a stable emulsion with respect to time^30^ before and after the demulsifier’s injection. The effectiveness of a demulsifier exists in the fact that it should be able to reduce the shear stress and viscosity of a highly stable crude oil emulsion^31^. As such, viscosity and shear stress characteristics of the formed emulsion containing either EC-4, EC-22 or EC-100 demulsifier were monitored with respect to time at 25 °C using the rheometer equipment. Figure 6a demonstrates the viscosity profile of crude oil emulsion before and after the addition of these demulsifiers. The viscosity profile of blank emulsion (without any demulsifier) was noticed to have remained unchanged throughout the entire 1200 s of the viscosity check. Following the injection of either EC-4, EC-22, or EC-100 demulsifier in the formed emulsions, a sharp drop in the emulsion viscosities was noticed. The viscosity reduction was more pronounced in the EC-4 demulsifier, suggesting that oil/water separation was faster than that of EC-22 and EC-100. These viscosity measurements corroborated the demulsification bottle test outcomes discussed in the previous section. Likewise, the shear stress (Fig. 6b) of emulsions containing the EC-4, EC-22 or EC-100 demulsifier dropped greatly as compared to that of blank emulsion which remained unchanged throughout the period of monitoring. The viscosity and shear stress profiles of these emulsions in the presence of EC-4, EC-22 and EC-100 demulsifiers indicated that they were able to travel to the oil–water interface and rupture the interfacial films.

**Figure 7:**
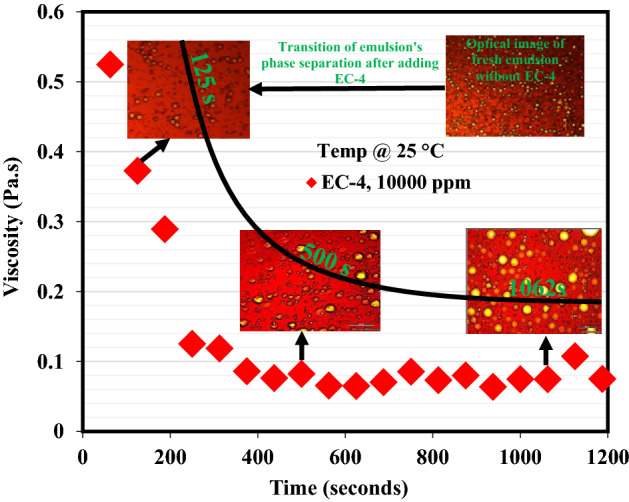
Optical microscopic images of emulsion containing EC-4 demulsifier. Images were taken after 125, 500, and 1062 s during the viscosity measurement.

**Figure 8:**
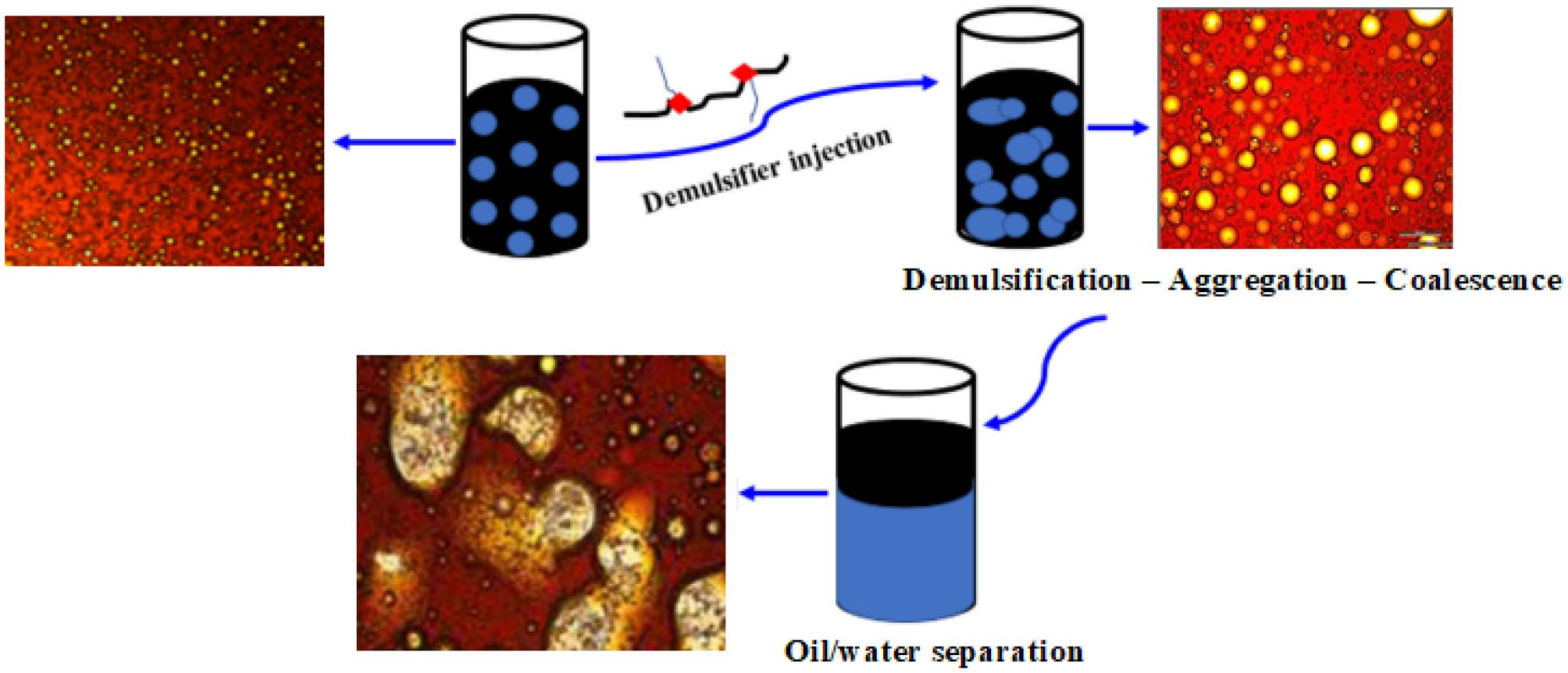
Proposed demulsification mechanism describing the demulsifying activity of an EC demulsifier.

To ascertain the rupture of oil–water interfacial films and the displacement of asphaltenes molecules, the emulsion containing EC-4 demulsifier was subjected to optical microscopic examinations during the viscosity measurement. Figure 7 shows the optical images of the systematic phase separation of emulsion filled with the EC-4 demulsifier at different periods during the viscosity measurement. At 125 s during measurement, the emulsion droplets were still small and nearly similar to the droplets of freshly prepared emulsion. As the emulsion viscosity dropped further, a few drops of this emulsion was carefully taken at 500 s and its image was examined under the optical microscope. The optical image at 500 s revealed that there was gradual aggregate and coalescence of emulsion droplets. Towards the end of emulsion viscosity determination, another few drops of the emulsion were analyzed under the microscope at 1062 s. From the optical microscopic image at this period, it is very glaring the emulsion droplets had formed larger aggregates and coalescences became more evident. Hence, the demulsification mechanism illustrating how these EC demulsifiers triggered demulsification, followed by water globules aggregation, coalescence, and systematic water/oil separation was described in Fig. 8.

## Conclusion

This study examined the demulsification performances of three ethyl cellulose (EC) demulsifiers with distinct viscosities. Crude oil emulsions were produced from either distilled water, seawater or various brines (NaCl, MgCl_2,_ and CaCl_2_). These EC demulsifiers have viscosities—4 cp, 22 cp, and 100 cp, and were designated as EC-4, EC-22, and EC-100 while their demulsification activity was conducted at ambient and elevated temperatures (25 °C and 90 °C). Demulsification experiments showed that EC-4 and EC-22 demulsifiers had a considerable demulsifying tendency in breaking crude oil emulsion formed from the distilled water at ambient temperature with %DE of 85.71% and 28.57%, respectively, while EC-100 demulsifier separated just 3.9% water at ambient condition. At an elevated temperature (90 °C), the three demulsifiers were able to break and remove water from the emulsion formed from distilled water. The %DE of EC-4, EC-22, and EC-100 was 99.23%, 58.57%, and 42.85%, respectively; while seawater accelerated the emulsion destabilization capacity of these demulsifiers at 90 °C. The demulsification performance of EC-4, EC-22, and EC-100 was estimated to be 99.23%, 58.57%, and 42.85%, respectively. Likewise, the demulsification performance of these demulsifiers was approximately the same (98%) in the presence of NaCl salt while MgCl_2_ and CaCl_2_ further accelerated the water removal efficiency of EC-22 and EC-100 by promoting their migration and adsorption at the oil–water interface. Rheological measurements showed that the injection of these demulsifiers in the formed emulsions led to a reduction in the shear stress and viscosity suggesting their migration, adsorption at the oil–water interface, and eventual collapse of asphaltene molecules. Future work should investigate how EC demulsifiers behave under different pH conditions. Future work would hopefully investigate how EC demulsifiers behave under different pH conditions and concentrations.

## Data Availability

The datasets used and/or analyzed during the current study are available from the corresponding author based on reasonable request.
